# Prevalence and associated factors of physical fighting among school-going adolescents in Namibia

**DOI:** 10.1186/1744-859X-6-18

**Published:** 2007-07-24

**Authors:** Emmanuel Rudatsikira, Seter Siziya, Lawrence N Kazembe, Adamson S Muula

**Affiliations:** 1 Departments of Epidemiology and Biostatistics and Global Health, School of Public Health, Loma Linda University, Loma Linda, CA, USA; 2 Department of Community Medicine, School of Medicine, University of Zambia, Lusaka, Zambia; 3 Department of Mathematical Sciences, University of Malawi, Chancellor College, Zomba, Malawi; 4 Department of Community Health, College of Medicine, University of Malawi, Blantyre, Malawi

## Abstract

**Background:**

Interpersonal physical violence is an important global public health concern that has received limited attention in the developing world. There is in particular a paucity of data regarding physical violence and its socio-demographic correlates among in-school adolescents in Namibia.

**Methods:**

We analysed cross-sectional data from the Namibia Global School-Based Health Survey (GSHS) conducted in 2004. We aimed to estimate the prevalence and socio-demographic correlates of physical fighting within the last 12 months. We obtained frequencies of socio-demographic attributes. We also assessed the association between self-reported history of having engaging in a physical fight and a selected list of independent variables using logistic regression analysis.

**Results:**

Of the 6283 respondents, 50.6% (55.2% males and 46.2% females) reported having been in a physical fight in the past 12 months. Males were more likely to have been in a physical fight than females (OR = 1.71, 95% CI (1.44, 2.05)). Smoking, drinking alcohol, using drugs and bullying victimization were positively associated with fighting (OR = 1.91, 95% CI (1.49, 2.45); OR = 1.48, 95% CI (1.21, 1.81); OR = 1.55, 95% CI (1.22, 1.81); and OR = 3.12, 95% CI (2.62, 3.72), respectively). Parental supervision was negatively associated with physical fighting (OR = 0.82, 95% CI (0.69, 0.98)). Both male and female substance users (cigarette smoking, alcohol and drug use) were more likely to engage in physical fighting than non-substance users (OR = 3.53, 95% CI (2.60, 4.81) for males and OR = 11.01, 95% CI (7.25, 16.73) for females). Parental supervision was negatively associated with physical fighting (OR = 0.85, 95% CI (0.72, 0.99)).

**Conclusion:**

Prevalence of physical fighting within the last 12 months was comparable to estimates obtained in European countries. We also found clustering of problem behaviours or experiences among adolescents who reported having engaged in physical violence in the past 12 months. There is a need to bring adolescent violent behaviour to the fore of the public health agenda in Namibia.

## Background

Interpersonal violence is an important global health problem. Physical fighting is one manifestation of interpersonal violence among adolescents [1]. In some countries, it is estimated that the economic burden of interpersonal violence amounts to an equivalent of at least 4%of the gross national product [2]. Interpersonal violence is ranked the fifth leading case of death among 15–44 year olds in the world [3]. The proportion of 13 year olds that report engaging in bullying once a week ranges from 1.2% in England and Sweden and 7.6% in the United States to 9.7% in Latvia [4]. Lai-Kah et al. reported that in 2001, 27.9% adolescents aged 12 to 19 years in Malaysia had been involved in a physical fight within the last 12 months preceding the survey [5]. There are limited data on the prevalence and predictors of interpersonal violence among adolescents in Africa. The available information on violence in Africa mostly concerns intimate partner violence, child soldiers, suicide, and sexual violence between adult males and females [6-9]. Among 604 randomly selected women attending antenatal care at King Edwards Hospital in South Africa, 52% reported physical violence from an intimate partner [10]. Lately there have been concerns that intimate partner violence is associated with HIV transmission [11].

In order to contribute to the literature on adolescent health behaviours, we carried out this study using existing data obtained from the Namibia Global School-based Health Survey (GSHS) conducted in 2004. Our study aimed to estimate the prevalence and associated factors of having engaged in a physical fight among school-going adolescents in Namibia. We believe knowledge about this estimate and associated factors will assist public health practitioners to establish programs, and policy makers and individuals involved in intervention programs could use the prevalence estimates to advocate for resource allocation for these programs. Identification of socio-demographic correlates of being engaged in physical fights may enable targeting of scarce resources to adolescents, who may be more vulnerable to physical fights and the associated consequences. The estimate may also allow cross-country comparisons regarding the prevalence of health behaviours and associated factors.

## Methods

Our study involved secondary analysis of existing data available from the Namibia Global School-Based Health Survey (GSHS), conducted in 2004. The GSHS was developed by the World Health Organization (WHO) in collaboration with UNICEF, UNESCO, and UNAIDS with technical assistance from CDC. The GSHS aims to provide data on health and social behaviours among school-going adolescents.

The GSHS used a 2-stage probability sampling technique. In the first stage, primary sampling units were schools that were selected with a probability proportional to their enrolment size. In the second stage, a systematic sample of classes in the selected school was obtained. All students in the selected classes were eligible to participate. A self-completed questionnaire was used.

For the main outcome, study participants were asked "During the past 12 months, how many times were you involved in a physical fight?" Eight options were provided, ranging from 0p-12 or more times. A response of 0 was described as not involved in a physical fight, while a response of ≥1 was classified as having engaged in a physical fight. Data analysis was performed using SUDAAN software (Research Triangle Institute, Durham, NC, USA, version 9.0).

A weighting factor was used in the analysis to reflect the likelihood of sampling each student and to reduce bias by compensating for differing patterns of non-response. The weight used for estimation is given by the following formula:

W = W1 × W2 × f1 × f2 × f3 × f4

Where W1 = the inverse of the probability of selecting the school, W2 = the inverse of the probability of selecting the classroom within the school, fl = a school-level non-response adjustment factor calculated by school size category (small, medium, large), f2 = a class-level non-response adjustment factor calculated for each school, f3 = a student-level non-response adjustment factor calculated by class, and f4 = a post stratification adjustment factor calculated by grade.

We obtained frequencies as estimation of prevalence of having engaged in a physical fight within the last 12 months. We also conducted logistic regression analysis to estimate the association between relevant predictor variables and physical fights. In addition, we conducted factor analysis to examine the intercorrelation of cigarette smoking, alcohol and drugs use. Then, we examined the relationship between those multiple behaviours together with physical fighting. We report unadjusted odds ratios and 95% confidence intervals for selected predictor variables, while considering suicidal thoughts as a dependent variable. We hypothesized that adolescents who had adequate parental supervision were less likely to be engaged in fights, and that females were less likely to be involved in fights. We also hypothesized that substance use (cigarette smoking, alcohol or drug use) analysed together will be associated the outcome of interest. We thereafter report results for adjusted odds ratios and 95% confidence intervals for the factors.

### Study setting

Namibia is a southern African country that shares borders with Angola and Zambia to the north, Botswana and Zimbabwe to the east, and South Africa to the south. The country is the second least densely populated country in the world, after Mongolia. Based on the 2001 population census, the country has a population of about 1.83 million [12]. At least 39% of the population is below the age of 15 years.

## Results

Table 1 presents selected characteristics of the study population of 6,283 Namibian adolescents (median age 14years old). Most of the sample was female (54.8%), 14 years old (24.7%), non-smokers (84.6%), non-alcohol drinkers (56.6%) and with parental supervision (50.6). Overall, 50.6% (55.2% males and 46.2% females) reported having been in a physical fight in the past 12 months.

**Table 1:** Socio-demographic characteristics of the study population

	Total % (n = 6283)	Males % (n = 2931)	Females % (n)
Age (years):	100 (6283)	45.2 (2931)	54.8 (3552)
≤13	22.2 (1474)	20.6 (616)	23.6 (858)
14	24.7 (1749)	23.1 (779)	25.9 (970)
15	23.2 (1471)	23.1 (648)	23.3 (787)
≥16	29.9 (1589)	33.1 (852)	27.3 (737)
Gender:			
Female	54.8 (3352)	-	-
Male	45.2 (2931)	-	-
Bullied:			
No	46.0 (2510)	42.5 (1080)	49.0 (1430)
Yes	54.0 (2470)	57.5 (1243)	51.0 (1227)
Substance use (cigarette smoking, alcohol or drug use	12.9 (699)	14.9 (389)	11.3 (310)
Parental supervision:			
No	49.2 (3045)	52.5 (1526)	46.9 (1519)
Yes	50.6 (3238)	47.5 (1405)	53.1 (1833)
Fighting:			
No	49.7 (3272)	44.8 (1323)	53.8 (1949)
Yes	50.6 (2919	55.2 (1573)	46.2 (1346)

Table 2 indicates that male subjects were more likely to be in a physical fight than females (OR = 1.43, 95% CI (1.26, 1.63)). Subjects who reported substance use (cigarette smoking, alcohol or drug use) were more likely to be in a physical fight than non-substance users (OR = 3.53, 95% CI (2.60, 4.81) for males and OR = 11.01, 95% CI (7.25, 16.73) for females). Subjects who smoked were more than three times as likely to be in a physical fighting than non-smokers (OR = 3.21, 95% CI (2.43, 4.24) for males and OR = 3.39, 95% CI (2.61, 4.40) for females). Those who reported drinking alcohol were twice likely to engage in physical fighting as those who did not (OR = 2.35, 95% CI (1.94, 2.83) for males and OR = 2.56, 95% CI (2.13, 3.08) for females). Bullying victimization was positively associated with physical fighting for both males and females (OR = 3.37, 95% CI (2.72, 4.17) for males and OR = 5.66, 95% CI (4.55, 7.04) for females). Subjects who had parental supervision were less likely to be in a physical fight than those who had no parental supervision (OR = 0.85, 95% CI (0.59, 0.95) for males and OR = 0.60, 95% (0.37, 0.85) for females).

**Table 2:** Physical fighting by age, gender, smoking, drinking alcohol, drug use, bulling victimization and parental supervision among adolescents in Namibia in 2004

	Unadjusted odds ratios with 95% CI		
	Total	Males	Females

Age (years):			
≤13	1.00	1.00	1.00
14	0.81 (0.68, 0.96)	0.84 (0.64, 1.10)	0.77 (0.61, 1.07)
15	0.92 (0.77, 1.10)	1.10 (0.84, 1.44)	0.79 (0.62, 1.01)
≥16	0.84 (0.70, 1.00)	0.77 (0.59, 1.00)	0.88 (0.68, 1.12)
Gender:			
Female	1.00	-	-
Male	1.43 (1.26, 1.63)	-	-
Substance use (cigarette smoking, alcohol or drug use):			
No	1.00	1.00	1.00
Yes	5.96 (4.67, 7.60)	3.53 (2.60, 4.81)	11.01 (7.25, 16.73)
Bullied:			
No	1.00	1.00	1.00
Yes	4.51 (3.88, 5.24)	3.37 (2.72, 4.17)	5.66 (4.55, 7.04)
Yes			
Parental supervision:			
No	1.00	1.00	1.00
Yes	0.78 (0.57. 0.99)	0.85 (0.59, 0.95)	0.60 (0.37, 0.85)

Table 3 presents results from multivariate analysis. Male gender, smoking, drinking alcohol, drug use and bullying victimization remained positively associated with physical fighting. Likewise, parental supervision remained negatively associated with physical fighting. In the factor analysis, the final communality estimate for cigarette smoking, alcohol and drug use was 0.92, which is an indication of high intercorrelation between the three variables.

**Table 3:** Physical fighting by age, gender, smoking, alcohol drinking, drug use, bulling victimization and parental supervision among adolescents in Namibia in 2004

Variable	Adjusted odds ratios with 95% CI
Age (years):	
≤13	1.00
14	0.87 (0.70, 1.08)
15	1.00 (0.80, 1.25)
≥16	0.82 (0.66, 1.03)
Gender:	
Female	1.00
Male	1.50 (1.28, 1.75)
Substance use (cigarette smoking, alcohol or drug use):	
No	1.00
Yes	4.12 (3.09, 5.50)
Bullied:	
No	1.00
Yes	3.67 (3.14, 4.30)
Parental supervision:	
No	1.00
Yes	0.85 (0.72, 0.99)

## Discussion

Our study found that the prevalence of having engaged in a physical fight among in-school adolescents in Namibia was 50.6% (55.2% for males and 46.2% for females). This estimate is about 1.5 times the prevalence reported by Lai-Kah et al. for Malaysian adolescents [5]. Our estimates however are much lower than those reported by Pickett et al. [1] in several countries in Europe, where prevalence was 53.3% in Wales and 58.2% in Austria. In virtually all settings where studies of physical fights have been conducted, males were more likely to be engaged in fights than females [1]. Research has suggested that traditional masculine gender socialization and social norms models encourage men to engage in behaviours that put their health at risk [13].

We found that having engaged in physical fighting was associated with cigarette smoking, alcohol and illicit drug use. Physical fighting was however negatively associated with parental supervision. Sosin et al. have reported that fighting behaviour could be one of the earliest and most reliable markers of multiple-risk-behaviour syndrome [14]. This clustering of unhealthy risk behaviours suggests that adolescents who are likely to engage in physical fights are also likely to be engaged in other risky behaviours.

It is also interesting to note that adolescents who reported having been bullied themselves were likely to have engaged in fights. The Global School-Based Health Survey did not collect data as to whether the study participant was the aggressor or was defending themselves in a physical fight. We are therefore unable to determine whether adolescents who reported being bullied were likely to initiate a fight or be dragged into a fight. Adolescents that have been victims of violence themselves may be at risk of being violent towards others. Rudatsikira et al. reported that in a multi-ethnic sample of adolescents in California, boys who had been victimized were more likely to carry weapons than those not previously victimized [15].

We also want to emphasise the role of parental support. We found that adolescents who reported parental support were 0.78% (95% CI 0.57, 0.99) as likely to be involved in fighting compared to those that did not report parental supervision. Springer et al. [16] have previously reported parental supervision as being associated with not only low aggression prevalence but also risky sexual behaviours. Parents need to be reminded of their role in supporting adolescents to become responsible citizens.

In the factor analysis, we found that both male and female substance users (cigarette smoking, alcohol and drug use) were more likely to engage in physical fighting those non-substance users.

Our study had several limitations. Firstly, data were collected through a supervised self-completed questionnaire. Some study participates may have misreported either wilfully or because of failure to recall. Recruitment of the study participants was also restricted to in-school adolescents. To the extent that in-school adolescents are different from adolescents outside school, our findings may not be applicable to all adolescents in Namibia. In addition, as data collection was cross-sectional, it was not possible to ascribe causation to any of the factors associated with the dependent variable.

## Conclusion

We found a 12-month prevalence of physical fighting among in-school adolescents of 50.6%, which is comparable to estimates obtained in Europe. The clustering of other problem behaviours or experiences such as cigarette smoking, alcohol and victimization from bullying suggests that public health intervention aimed at preventing adolescent interpersonal violence may have to factor in these other behaviours.

## Competing interests

The author(s) declare that they have no competing interests.

## Authors' contributions

ER conducted data analysis and participated in drafting of the manuscript. SS participated in interpretation of data and drafting of the manuscript. LNM participated in interpretation of data and drafting of the manuscript. ASM conceived data analysis plan, interpreted data and participated in drafting of the manuscript. All authors read and approved the final draft of the manuscript.
